# To look or not to look: Subliminal abruptonset cues influence constrained free-choice saccades

**DOI:** 10.16910/jemr.13.4.2

**Published:** 2020-07-20

**Authors:** Seema Prasad, Ramesh Mishra

**Affiliations:** University of Hyderabad, India

**Keywords:** Eye movement, eye tracking, saccades, subliminal cueing, attention, constrained free-choice, spatial ACS, abrupt-onset

## Abstract

Subliminal cues have been shown to capture attention and modulate manual response behaviour but their impact on eye movement behaviour is not well-studied. In two experiments, we examined if subliminal cues influence constrained free-choice saccades and if this influence is under strategic control as a function of task-relevancy of the cues. On each trial, a display containing four filled circles at the centre of each quadrant was shown. A central coloured circle indicated the relevant visual field on each trial (Up or Down in Experiment 1; Left or Right in Experiment 2). Next, abrupt-onset cues were presented for 16 ms at one of the four locations. Participants were then asked to freely choose and make a saccade to one of the two target circles in the relevant visual field. The analysis of the frequency of saccades, saccade endpoint deviation and saccade latency revealed a significant influence of the relevant subliminal cues on saccadic decisions. Latency data showed reduced capture by spatiallyirrelevant cues under some conditions. These results indicate that spatial attentional control settings as defined in our study could modulate the influence of subliminal abrupt-onset cues on eye movement behaviour. We situate the findings of this study in the attention-capture debate and discuss the implications for the subliminal cueing literature.

## Introduction

It is well known now that stimuli below the threshold of awareness can influence our responses [1, 2, 3]. While the effect of subliminal visual information on instructed responses is well studied [eg., 4, 5, 6], the susceptibility of voluntary eye movements to subliminal cues is not clear. Most laboratory tasks in cognitive psychological research involve responding to targets based on specific instructions. But, in our daily lives, we often choose between two or more alternatives freely, and under no specific constraints. The mechanisms involved in such “free” decisions and the factors influencing them has been an important topic of research [7]. Our interest was to examine if subliminal visual information can bias eye movement behaviour when participants make constrained free-choice saccades. Additionally, we also investigated if such influence is under strategic control induced by task-goals. The nature of control mechanisms at work when eye movements are triggered by subliminal cues is largely unknown. Can spatially-irrelevant subliminal cues be ignored such that they don’t influence eye movement behaviour (eg., not look at the notification light on the phone while reading a book)? We examined these questions using a variant of the spatial cueing paradigm [8] with subliminal abrupt-onset cues on a constrained free-choice saccade task.

In one of the first studies to show the influence of masked, subliminal cues on “free” responses, Schlaghecken and Eimer [9] asked participants to press the “left” key on seeing a left arrow, “right” key for a right arrow (forced-choice trials) and choose either of the keys for a double-headed arrow (free-choice trial). The visible targets were preceded by masked left/right arrows presented for 16 ms. On the free-choice trials, participants chose the key corresponding to the masked arrow more often than chance (in this case, 50%) suggesting that masked, subliminal visual information can influence responses even when they are freely chosen. Although it is close to two decades since this landmark study, there have only been a handful of studies examining the role of masked visual information on free choices (eg., 10, 11, 12, 13, 14) and specifically, free eye movements [15].

Eye movements are the primary mechanism through which we gather visual information and become aware of objects around us. But, what decides where we look? In line with previous models of oculomotor selection [16, 17]. Godjin and Theeuwes [18] proposed the concept of an oculomotor priority map in which saccade programming occurs through a single priority map where information from different sources is integrated. Thus, goal-relevance, physical salience and other factors compete together to drive eye movement behaviour. Godjin and Theeuwes [18] arrived at this model based on data that showed that participants were slower making a saccade to the target (a grey open circle among red open circles) when an additional irrelevant distractor (red open circle) was suddenly presented (far from the target) than when it was not. The exogenous signals from the sudden onset of the distractor interfered with saccade planning to the target thereby slowing it down. Thus, the oculomotor selection is determined by the integration of several competing programs and the resolution of this competition determines where the eyes land (but see 19 for an alternative oculomotor model). So far, factors such as selection-history, reward learning and emotion representations been shown to compete for selection in the oculomotor priority map and direct eye movements [20]. Our interest in this study was to see if subliminal cues can compete for selection in endogenously generated saccades and bias eye movements towards the location of the cues.

There are existing studies that demonstrate the influence of subliminal cues on saccade metrics [21, 22, 23]. But, most of these studies have used tasks where participants were instructed to make eye movements to specific locations. To our knowledge, there is only one study which has examined the influence of subliminal cues on free-choice saccades. Huang et al. [15] asked participants to freely choose to saccade to one of the four placeholders (white, horizontal Gabor patches) on the screen. Prior to the eye movement response, subliminal cues were presented for 33 ms at one or more locations. The cues were white vertically oriented Gabor patches which were rendered invisible by presenting a mask display (a grid of 25 white, horizontally oriented Gabor patches) for 260 ms preceding and following the cue. The authors observed that participants were more likely to saccade to the cued location compared to chance and were also faster when they did so compared to other locations. Since this is the only study so far to have shown such an effect, we wanted to re-examine it and also investigate the time-course of this effect.

Our additional interest was to examine if it is possible to strategically control the influence of subliminal cues on free eye movements. The relative contributions of stimulus-driven and goal-driven factors to the influence of subliminal cues have been a point of debate [see 24, 25, 26 for reviews]. Many researchers have argued that subliminal cues capture attention in a purely stimulus-driven manner - that is, irrespective of their relevancy to the current task [eg., 27]. Evidence for this comes from studies that show attention-capture by task-irrelevant cues. For instance, Weichselbaum et al. [23] presented white or black subliminal distractors (filled circles) while participants were asked to make a saccade to a white target (open diamond). The authors observed oculomotor capture by the cues (slower saccade latency to the target in the presence of a distractor) irrespective of whether they had the same (white cue - white target) or different contrast polarity (black cue - white target) compared to the target. On the other hand, some others have found that attention capture by subliminal cues is contingent on attentional control settings (ACS) generated by the task-goals [28, 29, 30]. While there is no eye movement study in support of this, Ansorge et al. [29] demonstrated top-down contingent capture by relevant masked cues on a task that required participants to search for a colour-defined target (eg., red) and discriminate based on its shape (diamond or square). The cues were colour singletons (eg., single green or red shape surrounded by three other red or green shapes) whose visibility was diminished by backward masking. The shape of the cue could either match or mismatch that of the target creating response congruency between the cue and the target. This was included with the additional goal of examining the extent of response activation by congruent cues which we won't discuss here. Importantly, location cue validity effects were seen only for the target-matching (red) but not for non-matching (eg., green) colour singleton cues lending support to the hypothesis that only goal-relevant masked cues capture attention.

Most studies with masked/subliminal peripheral cues have similarly manipulated relevancy based on feature match/mismatch between the cues and the targets where the task-relevance is established based on a feature such as colour, shape or onset type of the cues (along the lines of contingent-capture studies pioneered by Folk, Remington, and Johnston [31]; see Prasad and Mishra [26] for a detailed tabulation of such studies). But to our knowledge, no study so far has examined if spatial relevancy can modulate attention capture by brief, nearly-invisible cues. Although, the role of spatial ACS in modulating attention capture has been studied using *visible* cues [32, 33, 34]. Yantis and Jonides [34, Experiment 2], for instance, used black, central arrow cues which always indicated the direction of the target letter. Participants were equally fast in identifying the target (E or H) when the target was an abrupt-onset compared to when one of the distractors was an abrupt-onset. This showed that voluntary attention to a specific location can override exogenous attention capture (in this case, by the abrupt-onset distractor).

Similarly, Ishigami et al. [33] showed reduced attention capture by peripheral cues presented outside of the spatial ACS. They were also the first to demonstrate that multiple locations can be ignored (or attended-to) depending on the task-goals. Spatial ACS in this study was induced by instructing a group of participants to look for targets in the vertical visual field (in a display arranged in the form of a plus sign with four black-coloured figure-8 placeholders). Another group was assigned to the horizontal condition. The task was to identify a black coloured-digit (2 or 5) in the relevant visual field and press a key accordingly. Peripheral cues, created by brightening one of the figure-8 placeholders, were presented for 100 ms at the relevant-valid, relevant-invalid and irrelevant-invalid locations. Faster RT in the irrelevant-invalid condition compared to the relevant-invalid condition was seen suggesting that the irrelevant cues were not as efficient as relevant-invalid cues in capturing attention. These studies show that spatial attentional control settings can successfully modulate or even prevent capture by irrelevant peripheral cues.

### Current study

It is well-known that spatial and feature-based attention differ with regard to their time-course, strength and flexibility [35, 36, 37]. Thus, the role of top-down attention on subliminal processing should depend on which form of top-down attention is being manipulated [38, 26]. As mentioned before, no study so far has examined the role of spatial ACS with subliminal cues on free eye movements. On each trial, participants were instructed to make a single saccade to one of the two target circles in the upper or lower visual field. A central coloured circle indicated the relevant locations for that trial (eg., blue: up, green: down). These two types of trials appeared randomly. Thus, within the relevant visual field (eg., Up), participants were free to choose either of the two locations (eg., upper left or upper right). We will refer to this as “constrained free-choice” [see 39 for a similar terminology] to distinguish it from previous free-choice studies where there were no additional constraints on participants’ responses [eg., 15]. Prior to the saccade response, a subliminal cue was presented for 16 ms either in one of the two relevant locations or in one of the two irrelevant locations. The cues were expected to be masked from awareness due to the short presentation duration and the immediate display of placeholders following the cue. On a separate block of one-third of the trials, no cue was presented. We speculated that the sudden appearance of the cue on some trials might provide an alerting benefit irrespective of its location relevancy. Thus, to prevent the alerting mechanisms from confounding the orienting mechanisms triggered by the cue, cue absent trials were presented in a separate block.

We also manipulated the cue-target stimulus-onset asynchrony (SOA) to include three levels: 33 ms, 50 ms and 100 ms. Huang et al. (2014) did not manipulate SOA as they were presumably interested in obtaining the basic facilitatory effect of the subliminal cues. We wanted to replicate and extend their study by investigating the time course of these effects. The three levels of SOA were chosen based on the existing free-choice studies with manual responses that have observed facilitatory effects at short SOA and inhibitory effects at longer SOAs of a similar range [9, 13, 14]. Thus, we expected facilitation at 33ms and 50 ms SOA and inhibition at 100 ms SOA. The SOA condition was blocked because most studies examining the time-course of free-choice priming effects have used blocked SOA condition [9, 13, 14]. O'Connor and Neill [13] explicitly compared the effects of blocked vs. mixed SOAs on a free-choice priming study (Experiment 1a and 1b) using a design similar to Schlaghecken and Eimer [9]. They recommended blocking SOA in future research as it led to clearer effects of the masked primes on free-choice responses compared to the mixed SOA condition.

We measured the frequency, endpoint deviation, accuracy and latency of the saccades. First, in line with many previous studies, we expected the subliminal cues at relevant locations to influence saccades. We expected a higher frequency and faster latency of the saccades to the cued location. We also predicted the saccade end location to deviate more towards the cued location. Next, if it is possible to selectively filter out task-irrelevant information, irrelevant cues should not interfere with saccadic responses as much as relevant cues. As seen with Ishigami et al. [33], the responses on trials with irrelevant invalid cue should be faster compared to relevant invalid cue trials.

## Experiment 1

The sample size was determined using a power analysis (“pwr” package in R). The effect size was estimated to be from 0.5 to 0.9, based on previous research on subliminal cueing of eye movements where saccade latency was measured [15, 25, 40]. Cohen’s standardised difference scores (dz) were estimated using the reported paired-sample t-test values and sample sizes, i.e., dz = t/√N [41]. In the only study so far that has examined the influence of subliminal cues on the frequency of free saccades [15, one-cue condition], the sample size was 23 and the effect size 0.5. The power analysis yielded a sample size with a range of 11 to 33 with the desired power of 0.8 and a confidence level of 0.05. We selected a sample size that was within this range.

### Participants

Twenty-four participants (9 female, Mean age = 22.71 years, SD = 2.33) took part in the experiment. All participants reported normal or corrected-to-normal vision and provided written informed consent. All the procedures of this experiment and the subsequent experiments were approved by the Institutional Ethics Committee (IEC) of the University of Hyderabad.

### Apparatus

Experiment builder software (SR Research Ltd., Ontario, Canada) was used for stimuli presentation. Stimuli were presented in a dimly lit room on an LCD monitor with 1280 * 1024 resolution and 60 Hz refresh rate. Eye movement data were recorded using Eyelink 1000 desktop mount eye tracker with a sampling rate of 1000 Hz. A chin-rest and a forehead bracket were used to stabilise the head and maintain a viewing distance of 70 cm for all participants.

### Procedure

**Figure 1.**
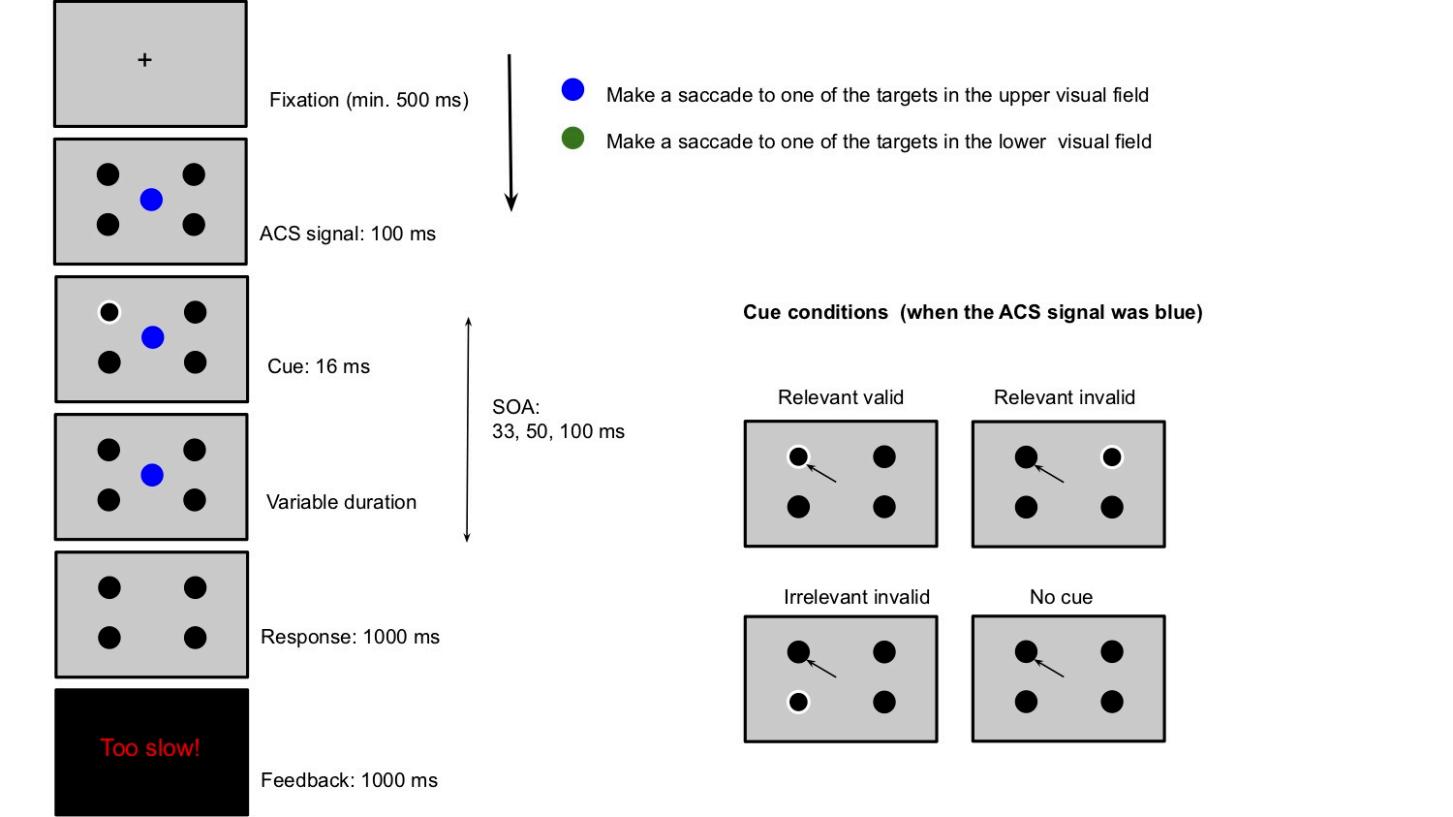
Experimental trial structure in Experiment 1. The ACS signal (blue central circle) shown in the example required the participants to make a saccade to one of the target circles in the upper visual field. In this example, the subliminal cue was presented in one of the relevant locations. The possible conditions as a function of cue-location and choice of saccade location are displayed on the right. The cue was not present during the eye-movement response. It is depicted so in the figure (on the right) to provide clarity on the experimental conditions. The same trial structure was used in Experiment 2. The only difference was that participants were asked to make a saccade to one of the target circles in the left or right visual field. Note: Only negative feedback were given: “Incorrect”, “Too slow” and “Trial aborted”.

The experiment began with a 9-point calibration. Each trial started with a fixation cross surrounded by four black filled circles of diameter 0.8° on a grey background (CIE-Lab: 63.33, 0.00, -0.00). A fixation trigger was used to ensure that the display did not move to the next screen unless the participant fixated on the cross for a minimum of 500 ms. The four circles were placed 7° from the centre and were equidistant from each other (Figure 1). This was followed by a central coloured circle of diameter 1° (blue CIE-Lab: 22.83, 58.29, -94.73 or green CIE-Lab: 37.08, -40.51, 41.55). The coloured circles henceforth referred to as “ACS signal” indicated the relevant visual field for the participants on that trial. For instance, a blue circle indicated that the participants had to make a saccade to one of the two circles in the upper visual field. The green circle indicated that one of the two circles in the lower visual field were the targets. The mapping between the colour of the ACS signal and the visual field was counterbalanced across participants. Next, the outline of one of the four black circles turned white for 16 ms, acting as the cue. The cue appeared in one of the two relevant locations on one-third of the trials and in one of the two irrelevant locations on the other one-third of the trials. The cue was equally likely to appear at each of the four locations. On the remaining one-third of the trials, no cue was presented. A display screen consisting of the central coloured circle and the four black circles was then presented for a variable duration of 17, 33 or 84 ms following which the ACS signal disappeared at which stage the participants were required to make their response. Thus, there were three levels of SOA between cue onset and the offset of the ACS signal: 33 ms, 50 ms, and 100 ms.

The participants were instructed to quickly look at one of the two black circles in the relevant visual field of the trial as soon as the ACS signal disappeared. They were given a maximum of 1000 ms to initiate the eye movement response, failing which they were prompted with a message “Too slow!” written in red (50 pt, Times New Roman) on a black background accompanied by a loud beep. The participants were also instructed to maintain fixation throughout the trial until the response. When eye movements away from the fixation were detected (before the response stage), the trial was aborted with a message written in red “Trial aborted” accompanied by a beep. An error message was similarly given if the saccades landed in a wrong (irrelevant) location. A blank screen was presented for 500 ms after every trial.

There were a total of 540 trials in the experimental session divided into two blocks: 360 trials with cue and 180 trials without the cue. This was the first level of blocking within which the trials were further divided equally between three levels of SOA (33, 50, 100 ms) and were presented in blocks. Within each SOA block, the trials were again divided equally between the two ACS signals (up and down) and presented randomly. The subliminal cues were presented in a relevant location half the times and in the irrelevant location half the times. Thus, in total there were 180 trials with a relevant cue and 180 trials with an irrelevant cue. Thus, the probability of the cue appearing was 25 % at each location. The trials in each block were presented randomly. The order of presentation of the blocks was counterbalanced across participants. The experiment lasted about 45 minutes with a break after every 120 trials. A practice session of 30 trials was administered first.

After the main experimental session, an objective visibility test was administered to assess the participants’ level of awareness of the cues. The same sequence of events as in the Main experiment was followed (only with relevant and irrelevant cues) except the participants were asked to identify the location of the subliminal cues. They were instructed to make a guess even if they were not sure about the cue’s location. A four-button (arranged in the form of a plus sign) Cedrus RB series response pad (SR research) was used to collect the responses. Participants pressed the button that was spatially congruent with the cue’s location.

A total of 150 trials were administered which consisted of 138 experimental trials + 12 control trials presented together. The experimental trials had 16 ms cue duration like in the main experiment; the control trials included cues of 500 ms duration. The objective of including control trials was to assess if the participants understood the instructions and performed accurately when the cues were clearly visible. The 150 trials were divided into three SOA blocks of 50 trials each. Each SOA block consisted of 25 trials with cues in relevant locations and 25 trials with cues in irrelevant locations. The order of presentation of the trials within each block and the blocks themselves was randomised.

### Data analysis

**Figure 2.**
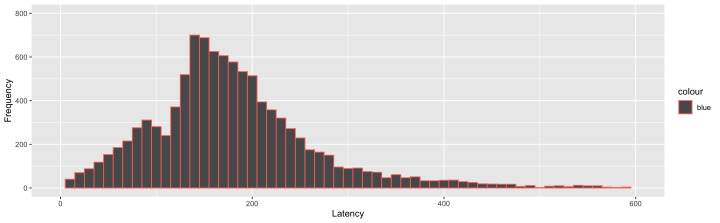
Frequency distribution plot of saccade latency in Experiment 1 showing two distinct peaks for express (80 ms - 130 ms) and regular saccades (> 130 ms)

On each trial, the first saccade that originated within an imaginary square of 4° width around the fixation cross and landed within 4° of four target circles was considered. Three participants’ data were discarded because more than 25 % of their saccades landed more than 4° away from the target circles. From the remaining participants’ data, 9 % of trials were discarded based on this criterion. Saccade latency was defined as the time taken to initiate a saccade following the disappearance of the ACS signal. Frequency distribution of saccades (Figure 2) revealed two distinct peaks which are considered as a hallmark of the presence of two types of saccades: express and regular saccades [42, 43]. Express saccades are those with a latency between 80 ms and 130 ms (15 % of the total saccades). Remaining saccades with the latency greater than 130 ms were considered as regular saccades. Saccades with latency less than 80 ms were discarded as being anticipatory (10.5 %). Separate analyses were performed on express and regular saccades. The upper limit for outliers among the regular saccades was calculated using the median absolute deviation (MAD) criterion as the more common method of discarding responses based on the standard deviation is considered to be not as effective in detecting outliers in smaller samples [44, 45]. Regular saccades with the latency greater than 2.5 MAD away from the median latency were discarded for each participant (10.7 %). On the remaining filtered trials, accuracy analysis was performed. Next, only those trials in which the saccades landed in the correct interest area (based on the spatial ACS of that trial) were considered for other analyses (88.28 %). The saccades could land in the relevant locations (correct saccades) or the irrelevant locations (categorised as errors). The saccades that landed correctly in one of the relevant locations could either be at the cued location or the opposite location.

Thus, trials were categorised into four types as a function of the location of the cue and the landing position of the saccade: relevant valid cue (cue location matches the correct saccade end location), relevant invalid cue (cue location opposite to the correct saccade end location, in the relevant visual field), irrelevant invalid cue (cue location in the irrelevant visual field) and no cue. The results from the analysis of regular saccades are reported below. The express saccades analysis lead to mostly non-significant results and are reported in the supplementary material.

Choice rate. The choice rate was calculated as the proportion of correct saccades to the cued location (relevant valid) and the opposite location (relevant invalid). Only relevant cue trials were included because participants could only choose one of the two relevant locations. To assess if the subliminal cues biased saccadic decisions when they were relevant, d’ was calculated on the choice rate (following the procedure of 11, 14] for each ACS signal type (Up and Down). For instance, for the ACS signal Up, the upper left cue was arbitrarily designated as the signal and the cue on the upper right was considered as noise. Hits referred to saccades that landed on the upper left quadrant when the cue was on the upper left. Saccades that landed on the upper left quadrant for the Upper right cue were considered as false alarms (FA). d’ prime was calculated as the difference in z transformed values of hit rates and false alarms. d’ was similarly calculated for ACS signal Down. Hits and false alarm rates of 0 or 1 were corrected using the log-linear rule [46]. A t-test was performed comparing the mean d’ value with chance (0). Next, to examine the effect of SOA and ACS signal, we constructed a linear mixed-effects model using the lmer function. The effect of SOA was analysed by constructing two columns: SOA100_50 (SOA 100 ms: +1, SOA 50 ms: -1) and SOA100_33 (SOA 100 ms: +1, SOA 33 ms: -1). ACS signal was also sum coded (Up: -1, Down: +1). All these factors were entered as fixed effects.

Accuracy. Our objective was to compare the accuracy level across relevant (R), irrelevant (IR) and no-cue trials. It was not possible to compare the accuracy between relevant valid and relevant invalid saccades here because this was a constrained free-choice task where a saccade to either of the relevant locations (valid or invalid) was considered correct. The difference between the frequency of correct saccades to relevant valid and relevant invalid is captured in the choice rate analysis described above. In the accuracy analysis, we primarily wanted to examine the accuracy in following the ACS signal as a function of cue type. d’ was calculated for accuracy. Correct responses to ACS signal Up (saccades to one of the upper locations) were considered as Hits and Incorrect responses to ACS signal Down (saccades to one of the upper locations) were considered as false alarms. “Relevancy” was included as a fixed effect in the analysis by creating two columns. One column compared irrelevant cue- and no-cue trials (IR_No; +1: irrelevant cue, -1: no cue) and another column compared the relevant- and no-cue trials (R_No; +1: relevant cue, -1: no cue). SOA100_33, SOA 100_50 and their interactions with relevancy were also added as fixed effects.

Saccade endpoint deviation. The mean horizontal deviation of the saccade landing position from a central vertical line was calculated in degrees [following 47]. For each ACS signal, the endpoint deviation was calculated such that saccades landing to the right of the central line had a positive sign, those landing to the left had a negative sign. Thus, a positive (rightward) deviation in the presence of a right cue and a negative (leftward) deviation in the presence of a left cue would indicate that the subliminal cues have facilitated the saccade endpoint deviation. In contrast, a positive deviation in the presence of a left cue and a negative deviation in the presence of a right cue would indicate inhibition of the cued location. Trials with relevant and irrelevant cues were analysed separately. Mixed-effects analysis was performed with cue location (left: -1, right: +1), SOA (SOA100_33 and SOA50_33), ACS signal and their interactions as fixed effects.

Saccade Latency. Mixed-effects analysis was performed on saccade latency using the lmer function. There were three levels of the “Condition” variable: relevant valid, relevant invalid, and irrelevant invalid. No cue condition was not included in the analysis as it lacks the alerting component involved in all the other conditions. Thus, it differed from the other conditions not only in terms of orienting but also in terms of alerting. The effect of condition was analysed by creating two columns: conditionRIV_RV (relevant invalid: +1, relevant valid: -1) and conditionRIV_IR(relevant invalid: +1, irrelevant invalid: -1). SOA100_33, SOA 100_50, ACS signal and their interactions with condition were also added as fixed effects.

In all the analyses involving d’ (frequency and accuracy), Participants was entered as a random effect. For the latency and endpoint deviation analyses, both Participants and Items were included as random effects. All main effects and interactions were included for each model. lmerTest function was used to obtain p values using Satterthwaite approximations to degrees of freedom [48]. The description of the final model and the output for all the analyses is given in the Appendix.

Cue localisation. Trials with response times greater than 130 ms were discarded from analysis (3.4 %). Accuracy on experimental and control trials was calculated. Cue visibility index was calculated by creating pairs of cue locations: Q1 - Q2, Q1 - Q3, Q1 - Q4, Q2 - Q3, Q2 - Q4, Q3 - Q4 (Q1, Q2, Q3, Q4 represent each of the four quadrants). For each pair, the cue appearing at one location (for eg., Q1) was arbitrarily designated as the signal and the cue appearing at the other location (for eg., Q2) was designated as noise. Correct responses to the signal (selecting Q1 when the cue appeared in Q1) were considered as Hits and incorrect responses to the noise (only those responses that were same as the correct response to the Hits, for a given pairing - for example, selecting Q1 when the cue appeared in Q2) were considered as false alarms (FA). Hits and FAs were corrected using the log-linear rule to adjust for occurrences of Hits and FAs of 0 or 1 [46, 14]. Hit rate and FA rate were calculated by dividing the Hits and FAs by the total number of signal and noise trials, respectively. The Hit rates and FA rates across the 6 pairs of cue locations (Q12, Q13, Q14, Q23, Q24, Q34) were averaged to yield a single Hit and FA rate. The mean d’ was computed as the difference of the z transform of the mean Hit rate and the mean FA rate using the norm function in R. One-sample t-tests were conducted to examine if the cue visibility significantly differed from chance (0). Paired sample t-tests were conducted to compare the visibility between cues at relevant and irrelevant locations.

Additionally, we calculated Bayes factors to supplement the results as frequentist statistics is not appropriate for accepting a null hypothesis - that the performance on cue visibility test is equivalent to the chance level. Bayesian hypothesis testing involves setting up two models with two contrasting hypotheses and adjusting the likelihood of each model based on the evidence (data obtained). We tested for model H0 defined as cue visibility being same as the chance level against model H1 defined as better cue visibility compared to chance level. We performed one-sample t-tests using JASP comparing d’ with chance level (0). Bayes factors (BF01) were computed which quantify the relative evidence for the two competing hypotheses. According to a commonly accepted convention [49, 50], 3 > BF01 > 1 denotes anecdotal evidence, 10 > BF01 > 3 denotes moderate evidence and BF01 > 10 denotes strong evidence *for* the null hypothesis. Similarly, 1/3 < BF01 < 1 denotes anecdotal evidence, 1/10 < BF01 < 1/3 denotes moderate evidence and BF01 < 1/10 denotes strong evidence for the alternate hypothesis. BF01 = 1 suggests that the data is inconclusive.

## Results

**Figure 3.**
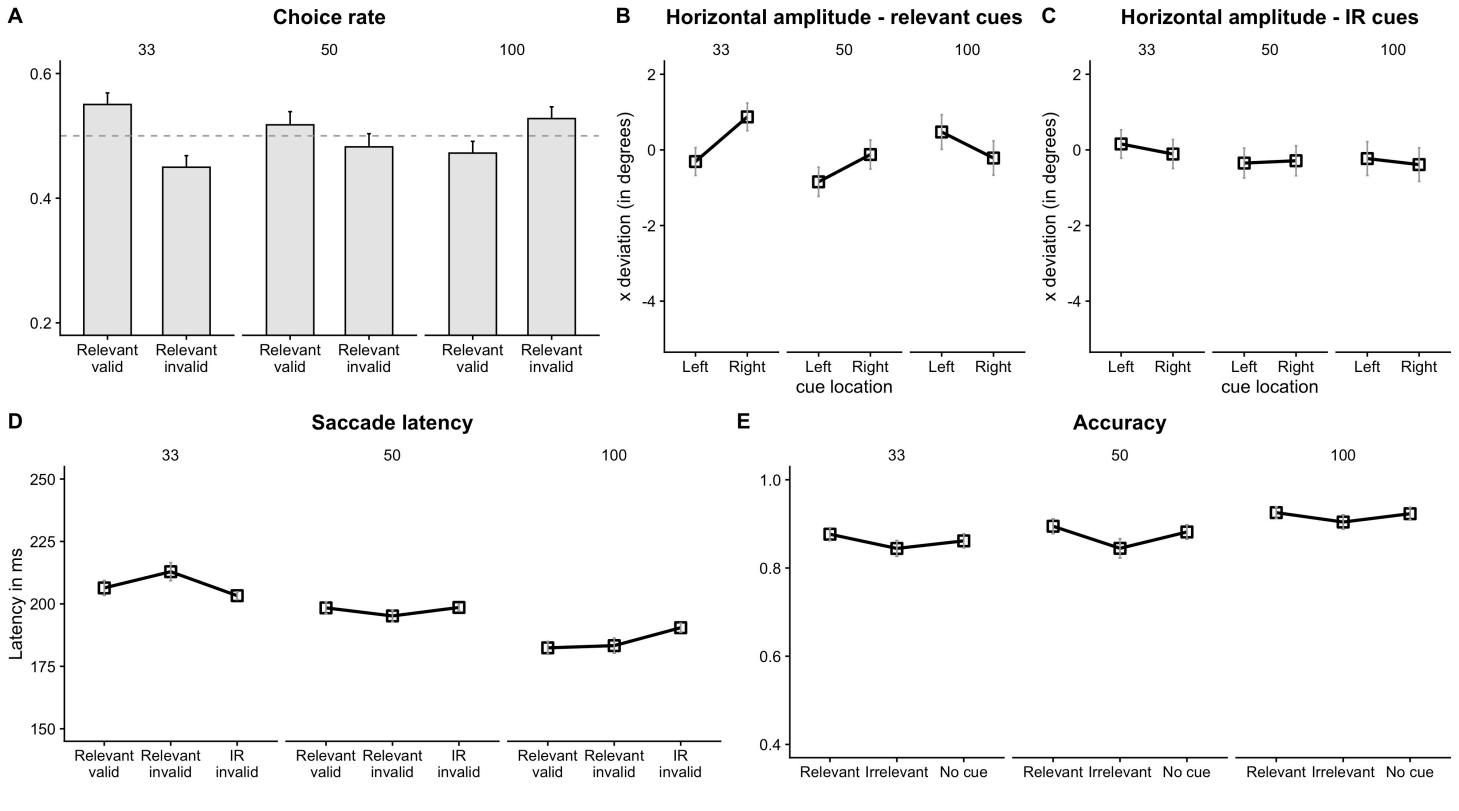
Results from Experiment 1 (A) There were more saccades to the relevant valid location at 33 ms and 50 ms SOA compared to relevant invalid location (B) The horizontal deviation of the saccade end location showed that saccades landed towards the cued locations at 33 ms and away from the cued locations at 100 ms SOA, only when the cues were relevant (C) but not when they were irrelevant (D) Facilitation followed by inhibition was observed in latency of saccades to the (relevant) cued location. But latency on IR invalid cue trials was faster compared to relevant invalid trials at 33 ms. This pattern was reversed at 100 ms SOA (E) Participants were less accurate on IR cue trials compared to no cue trials, accuracy on relevant cue trials was also greater compared to no cue. Note: The choice-rate and accuracy data are plotted in terms of proportion for ease of comprehension. The statistical analysis was performed on d’ values. The dashed horizontal line represents chance level (0.5)

Choice rate. Overall d’ prime was significantly greater than chance level suggesting that saccades landed at the location of the relevant cue more often than the opposite location, *t* (1, 20) = 2.02, *p* = 0.057. Mixed-effects analysis revealed a significant effect of SOA on d’ (SOA 100 ms vs. SOA 33 ms: β = -0.19, *t* = -2.37, *p* = 0.019). The proportion of saccades to the cued location (see Table 1 for means and Figure 3A) were much higher at 33 ms SOA compared to 100 ms SOA. Separate t-tests were performed on d’ at each SOA. d’ was significantly greater than chance at 33 ms, *t* (1, 20) = 2.4, *p* = 0.026, but not at 50 ms SOA , *t* (1, 20) = 1.22, *p* = 0.236 and 100 ms SOA, *t* (1, 20) = -1.28, *p* = 0.215. There was no effect of ACS signal, β < 0.001, *t* = 0.01, *p* = 0.994, suggesting that the influence of relevant cues did not vary as a function of whether participants were told to look up or down. The interactions between ACS signal and SOA were not significant either (*t* < 1.5).

Saccade endpoint deviation (horizontal amplitude). There was no main effect of (relevant) cue location, β = 0.23, *t* = 1.43, *p* = 0.15 (Figure 3B). There was a significant interaction between cue location and SOA at 100 ms compared to 33 ms, β = - 0.49, *t* = - 2.05, *p* = 0.041. Saccades landed towards the location of the cue at 33 ms SOA (leftward deviation for left cues and rightward deviation for right cues). This effect was reversed at 100 ms SOA (leftward deviation for right cues and rightward deviation for left cues) which indicated that the cued locations were inhibited. There was a significant main effect of ACS signal, β = 0.54, *t* = 3.37, *p* < 0.001, indicating greater positive (rightward) deviation whenever participants were instructed to look Up, irrespective of the cue location.

Spatially- irrelevant cues did not affect the saccade endpoint deviation, β = -0.01, *t* = - 0.08, *p* = 0.938 (Figure 3C). The interaction between irrelevant cue location and SOA was also not significant either, 100 ms: β = - 0.05, *t* = - 0.23, *p* = 0.821; 50 ms: β = - 0.05, *t* = - 0.23, *p* = 0.817.

Saccade Latency. Latency on relevant valid and relevant invalid trials across SOAs were comparable, β = 1.17, *t* = 0.98, *p* = 0.326 (Figure 3D). However, participants were faster on relevant valid trials compared to relevant invalid trials at 33 ms as indicated by a significant interaction between SOA and condition (100 ms vs 33 ms: β = 3.84, *t* = 2.14, *p* = 0.032). This pattern was also reversed at 50 ms which was significant compared to 33 ms SOA (50 ms vs 33 ms: β = -3.21, *t* = -1.97, *p* = 0.049). There were no overall differences in latency between IR invalid cue trials and relevant invalid trials, β = -1.16, *t* = -1.14, *p* = 0.255 either. There was a significant interaction between SOA and IR invalid cue condition (100 ms vs 33 ms), β = -6.73, *t* = -4.43, *p* < 0.001. Participants were faster in making saccades in the IR invalid condition compared to relevant invalid condition at 33 ms, but the pattern was reversed at 100 ms SOA (see Table 1 for means). Saccades were faster when the instruction was to look up vs. down as indicated by a main effect of ACS signal, β = 1.59, *t* = 1.97, *p* = 0.049. Saccades were also faster on trials with 100 ms SOA compared to 33 ms SOA (100 ms vs 33 ms: β = -12.41, *t* = -10.27, *p* < 0.001). None of the other interactions were significant (*t* < 1.3).

**Table 1.** Descriptive statistics (Experiment 1)

	Choice rate (relevant valid)	End location (horizontal amplitude)			Saccade latency		
		Left cue	Right cue	Relevant valid	Relevant invalid	IR invalid	No
33 ms	0.55 (0.08)	-0.31 (7.18)	0.87 (7.28)	206 (61)	213 (68)	203 (56)	203 (60)
50 ms	0.52 (0.09)	-0.84 (7.39)	-0.12 (7.44)	198 (48)	195 (45)	198 (48)	199 (57)
100 ms	0.49 (0.08)	0.47 (7.5)	-0.21 (7.47)	182 (41)	183 (50)	196 (65)	184 (48)

Note: Latencies are given in ms. Horizontal amplitude of endpoint deviation is given in degrees. Numbers in bracket denote +1 SD. Since the choice rate on relevant-valid and relevant-invalid trials is complementary, values are given only for relevant-valid trials. IR: irrelevant.

Accuracy. Participants were less accurate on IR cue trials (*M* = 86 %, *SD* = 9) compared to no cue trials (*M* = 89 %, *SD* = 7), β = -0.12, *t* = -2.26, *p* = 0.025 (Figure 3E). The accuracy on relevant cue (*M* = 90 %, *SD* = 7) trials was also greater compared to no cue trials, β = 0.14, *t* = 2.48, *p* = 0.014. There was also a main effect of SOA indicating greater accuracy on 100 ms SOA trials (*M* = 92 %, *SD* = 7) compared to 33 ms SOA trials (*M* = 86 %, *SD* = 7), β = 0.21, t = 3.89, *p* < 0.001.

We next examined if there were more error saccades to the location of the irrelevant cue as opposed to the opposite location. The overall d’ value across SOA was not significantly different from chance *t* (1, 20) = 0.25, *p* = 0.804. A mixed effects analysis on the d’ values revealed a nonsignificant effect of SOA (50 ms vs 33 ms: β = -0.08, *t* = -0.77, *p* = 0.443; 100 ms vs 33 ms: β = -0.01, *t* = -0.12, *p* = 0.904).

Cue localisation. The accuracy on control trials (*M* = 59.1 %, *SD* = 30) and experimental trials (*M* = 40.8 %, *SD* = 18.1) were both significantly different from chance level (25 %), *t* (1, 20) = 5.21, *p* < 0.001 and *t* (1, 20) = 3.99, *p* = 0.001 respectively. The cue visibility index (*M* = 0.5, *SD* = 0.77) was also significantly greater than zero, *t* (1, 20) = 2.97, *p* = 0.007. Paired t -tests revealed no significant difference between the visibility for cues at relevant (*M* = 0.52, *SD* = 0.7) and irrelevant locations (*M* = 0.46, *SD* = 0.9), *t* (1, 38.51) = -0.21, *p* = 0.832.

Bayesian statistics showed that there was moderate evidence to accept the alternate hypothesis (BF01 = 0.16).

## Discussion

The subliminal cues had a significant influence on saccadic decisions when they were spatially-relevant. The frequency of saccades to the cued location was greater than the saccades to the opposite location. We also observed significant leftward deviation in the saccade endpoint for cues on the left and rightward deviation for cues on the right at 33 ms suggesting that the cues facilitated the saccade endpoint deviation at short SOA. This pattern was reversed at 100 ms SOA indicating inhibition of the cued location. Participants were also faster making saccades on relevant valid trials, but only at the short SOA (33 ms). All these results indicate that we found the classic pattern of facilitation at short SOA followed by (not so significant) inhibition at longer SOA commonly observed in studies with peripheral cues [51]. Thus, we found converging results to conclude that spatially-relevant cues biased both the spatial and temporal characteristics of constrained free-choice saccades in a manner similar to the influence on instructed responses.

We had hypothesised that if the participants can filter out spatially-irrelevant cues to some extent, then the participants should be faster on IR invalid cue trials compared to relevant invalid trials. We found exactly that at 33 ms SOA. The pattern was reversed at 100 ms in line with the inhibitory effects seen with relevant cues. Thus, the trials with IR invalid cues captured less attention than relevant invalid trials suggesting that participants were less influenced by IR cues. Interestingly, participants made more errors on IR-cue trials compared to no-cue trials indicating that IR cues did influence saccadic responses. Thus, we can only conclude that while participants could not completely ignore the IR cues, there was reduced capture by IR cues.

## Experiment 2

We can conclude two things from Experiment 1: First, subliminal cues influenced saccadic decisions. Second, there was reduced capture by spatially-irrelevant cues. In Experiment 2, we sought to replicate these findings with a slightly different design. It is well known that spatial biases play a role in determining eye movement behaviour [52, 53, 54, 55]. To examine if the observed findings could be generalised and was not restricted to the way we defined the ACS (up vs. down), we conducted another experiment with a different type of ACS (left vs right). Participants were asked to choose one of the target locations on the left or right depending on the ACS signal at the centre. Based on the results of Experiment 1, we expected 1) the subliminal cues to similarly influence frequency of saccades, endpoint deviation and latency when the cues were spatially relevant 2) the latency on IR invalid cue trials to be faster than relevant invalid trials at short SOA 3) accuracy on IR cue trials to be worse compared to no cue trials.

### Participants

Twenty-two individuals (19 males, Mean age = 25.27 years, SD = 3.19) took part in the experiment. None of them had participated in the previous experiment.

### Procedure

The apparatus and stimuli were exactly same as Experiment 1. The procedure was similar to that of Experiment 1, except the participants were asked to choose one of the two targets in the left and right. Thus, blue and green circles indicated Left and Right direction respectively for half of the participants (and vice-versa for other half of the participants). The number of trials and the blocking levels were same as Experiment 1.

### Data analysis

The analysis procedure was similar to that of Experiment 1. Four participants’ data was discarded from final analysis because more than 25% of the saccades landed more than 4° away from the target circles (irrespective of the ACS). From the remaining participants’ data, 10 % of the trials were discarded based on this criterion; 18 % saccades were discarded for being anticipatory. 16.5 % of trials were express saccades (130 ms > latency > 80 ms, Figure 3) and were analysed separately (Supplementary material). Among the regular saccades, 10.6 % of the trials were discarded as outliers based on the upper limit determined by MAD criteria. Error trials constituted 10.25 % of the trials. In the cue visibility test, 6% of the trials were discarded as outliers (RT < 130 ms)

**Figure 4.**
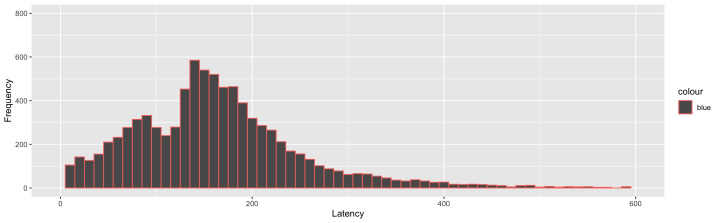
Frequency distribution plot of saccade latency (Experiment 2) showing two distinct peaks for express (80 ms - 130 ms) and regular saccades (> 130 ms)

In the choice rate analysis, d’ was calculated for each ACS signal (Left and Right). For instance, for ACS signal Left, saccades to the upper left quadrant on trials with upper left cue were considered Hits. Saccades to the upper left quadrant on trials with lower left cue were considered False Alarms. d’ was similarly calculated for ACS signal Right. Mixed effects analysis was done on d’ values with ACS signal (Left: -1, Right: +1) and SOA (SOA100_33, SOA 100_50) as factors. In the saccade endpoint deviation analysis, vertical amplitude was calculated. This was because the ACS signals in Experiment 2 were Left or Right. For each type of signal, the cue could be located either up or down. The deviation of the saccade landing position (vertical amplitude) from a central horizontal line was calculated. A positive number indicated a downward deviation and a negative number indicated an upward deviation compared to the central horizontal line. Mixed effects analysis on saccade endpoint deviation was performed with cue location (up: -1, down: +1), ACS signal and SOA as fixed effects. For the accuracy analysis, d’ prime was calculated by considering correct (leftward) responses to ACS signal Left as Hits and incorrect (leftward) responses to ACS signal Right as False Alarms. Relevancy (IR_No and R_No) and SOA were entered as fixed effects. Saccade latency was analysed by entering condition (conditionRIV_RV and conditionRIV_IR), SOA and ACS signal as factors into the mixed-effects model.

## Results

**Figure 5.**
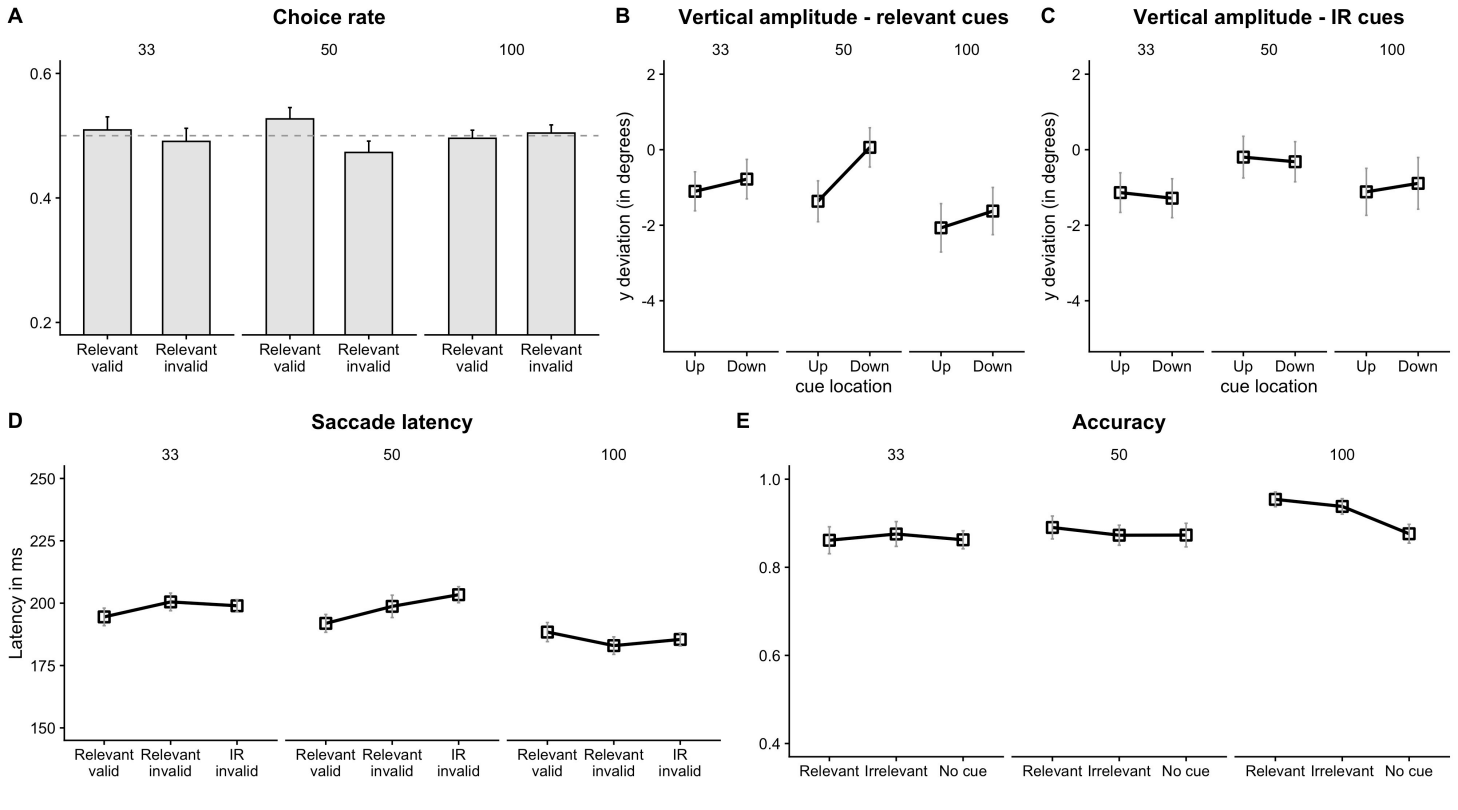
Results from Experiment 2 **(**A) Frequency of saccades to the cued location was significantly greater than chance at 50 ms SOA. (B & C) The vertical deviation of the saccade end location was relatively more downward (more positive) for “down” cues compared to “up” cues at all SOAs, only when the cues were relevant (D) Relevant cues facilitated saccade latency at 33 ms and 50 ms SOA which turned into inhibition at 100 ms SOA. Saccades on trials with IR invalid cues were faster compared to relevant invalid trials at 33 ms SOA. This pattern was reversed at 50 ms (E) No differences in accuracy were found across different cue conditions. Note: The choice-rate and accuracy data are plotted in terms of proportion for ease of comprehension. The statistical analysis was performed on d’ values. The dashed horizontal line represents chance level (0.5)

Choice rate. The frequency of saccades to the relevant valid location was not significantly greater than chance, *t* (1, 17) = 1.45, *p* = 0.165. lmer analysis on d’ showed that SOA did not significantly modulate the frequency of saccades to the relevant valid location, (50 ms vs. 33 ms: β = 0.12, *z* = 1.6, *p* = 0.113; 100 ms vs. 33 ms: β = - 0.11, *z* = -1.39, *p* = 0.152) (Figure 5A). Individual t tests on d’ values were performed separately at each SOA. Saccades to the relevant valid location were greater than chance at 50 ms, *t* (1, 17) = 2.17, *p* = 0.044 but not at 33 ms SOA, *t* (1, 17) = 0.35, *p* = 0.733 and 100 ms SOA, *t* (1, 17) = -1.39, *p* = 0.185 (see Table 2 for descriptive means). There was no effect of the ACS signal (β = - 0.04, *z* = -0.67, *p* = 0.506) suggesting that the instruction to look Left or Right did not modulate the influence of the cues on choice rate.

**Table 2.** Descriptive statistics (Experiment 2)

	Choice rate (relevant valid)	End location (horizontal amplitude)			Saccade latency		
		Up cue	Down cue	Relevant valid	Relevant invalid	IR invalid	No
33 ms	0.51 (0.09)	-1.1 (8.78)	-0.78 (8.77)	194 (58)	200 (59)	198 (58)	204 (57)
50 ms	0.53 (0.08)	-1.36 (8.75)	0.06 (8.94)	192 (61)	199 (72)	203 (75)	204 (59)
100 ms	0.49 (0.05)	-2.07 (8.92)	-1.62 (8.79)	188 (54)	183 (48)	185 (50)	180 (41)

Note: Latencies are given in ms. Horizontal amplitude of endpoint deviation is given in degrees. Numbers in bracket denote +1 SD. Since the choice rate on relevant-valid and relevant-invalid trials is complementary, values are given only for relevant-valid trials. IR: irrelevant-cue.

Saccade endpoint deviation (vertical amplitude). There was a main effect of cue location, β = 0.40, *t* = 2.05, *p* = 0.041 (Figure 5B) suggesting that the deviation from a central horizontal line was relatively more positive (downward) for Down cues compare to Up cues. This indicates an overall facilitatory effect of the cues on saccade deviations. This facilitatory effect was greater at 50 ms SOA compared to 33 ms SOA, β = 0.53, *t* = 1.97, *p* = 0.049 as indicated by a significant interaction between cue location and SOA. There was a significant three-way interaction between ACS signal, cue location and SOA, β = -0.7, *t* = -2.37, *p* = 0.018. Separate models on data corresponding to each ACS signal showed that SOA and cue location interaction was present only for the ACS Left condition (50 ms vs 33 ms: β = 09.92, *t* = 2.44, *p* = 0.015; 100 ms vs 33 ms: β = -1.12, *t* = -2.8, *p* = 0.005), but not for ACS Right condition (50 ms vs 33 ms: β = 0.04, *t* = 0.12, *p* = 0.907; 100 ms vs 33 ms: β = 0.31, *t* = 0.71, *p* = 0.479). There was a main effect of ACS signal, β = 0.85, *t* = 4.28, *p* < 0.001 indicating that saccade deviation was more downward for ACS signal Right compared to Left.

Spatially irrelevant cues did not influence saccade endpoint deviation, β = 0.02, *t* = 0.11, *p* = 0.910 (Figure 5C). The interactions with SOA or ACS signal were not significant either (*p* > 0.5)

Saccade Latency. Saccade latency on the relevant valid cue trials was lesser compared to the relevant invalid cue condition, β = 3.44, *t* = 2.46, *p* = 0.014 (Figure 5D). This effect was marginally greater at 50 ms SOA compared to 33 ms SOA, β = 3.38, *t* = 1.77, *p* = 0.078. The facilitatory effects observed at 33 ms and 50 ms SOA turned into inhibition at 100 ms as revealed by a significant interaction between SOA and condition (100 ms vs 33 ms), β = -4.56, *t* = -2.17, *p* = 0.03. The latency on irrelevant invalid cue trials was faster compared to the latency on relevant invalid cue trials, β = -2.3, *t* = -1.9, *p* = 0.058. This pattern was observed at 33 ms SOA, but the opposite pattern was observed at 50 ms SOA (50 ms vs 33 ms: β = -3.28, *t* = -1.98, *p* = 0.048). There was no main effect of ACS signal, β = -1.23, *t* = - 1.28, *p* = 0.199. It did not interact with any of the conditions either (t *<* 1.3). Shorter overall latencies were observed on trials with 100 ms SOA compared to 33 ms SOA, β = -15.85, *t* = -10.81, *p* < 0.001. Latency on 50 ms SOA trials was greater compared to 33 ms SOA, β = 7.37, *t* = 5.58, *p* < 0.001.

Accuracy. There was no difference in accuracy between the relevant cue (*M* = 90 %, *SD* = 11) and no cue trials (*M* = 87 %, *SD* = 9.6), β = -0.09, *z* = 1.2, *p* = 0.232. The accuracy on IR cue trials (*M* = 89 %, *SD* = 10) was also comparable to no-cue trials, β = 0.01, *z* = 0.20, *p* = 0.841 (see Table 2 for condition-wise mean values and Figure 5E). SOA did not modulate accuracy on either of these types of trials (100 ms vs 33 ms: β = -0.01, *t* = -0.14, *p* = 0.887; 50 ms vs 33ms: β = 0.04, *t* = 0.6, *p* = 0.55).

The analysis on error saccades revealed that the number of error saccades at the location of the irrelevant cue was comparable to the other location, *t* (1, 17) = -0.54, *p* = 0.593. SOA did not have any influence either (100 ms vs 33 ms: β = 0.04, *t* = 0.48, *p* = 0.634; 50 ms vs 33ms: β = -0.02, *t* = -0.21, *p* = 0.835). Separate t tests performed at each SOA revealed nonsignificant differences compared to chance (*p* > 0.5).

Cue localisation. Participants performed significantly better than chance level (25 %) on control trials (*M* = 43.98 %, *SD* = 30), *t* (1, 17) = 2.67, *p* = 0.016. Accuracy on the experimental trials (*M* = 33.58 %, *SD* = 17.4) was marginally greater than chance level performance, *t* (1, 17) = 2.09, *p* = 0.052 respectively. The cue visibility index (*M* = 0.31, *SD* = 0.72), even though very small, was significantly different from zero, *t* (1,17) = 1.83, *p* = 0.085. The visibility did not differ significantly between the cues at relevant (*M* = 0.17, *SD* = 0.7) and irrelevant locations (*M* = 0.38, *SD* = 0.73), *t* (1, 33.94) = 0.86, *p* = 0.393.

Bayesian hypothesis testing revealed anecdotal evidence to accept the null hypothesis (BF01 = 1.04).

## Discussion

We conducted Experiment 2 to verify if the findings of Experiment 1 could be generalised across different types of spatial ACS. Participants made more saccades to the cued location at one of the shorter SOAs (50 ms). The vertical amplitude of the saccade endpoint deviation was also influenced by the relevant cues. The “up” cues lead to more relatively upward deviation than the “down” cues and vice versa. This effect was also greater at 50 ms SOA compared to 33 ms SOA. The interaction with SOA was only observed for ACS signal Left, but not for ACS signal Right. Latency on relevant valid trials was faster than relevant invalid trials at the shorter SOAs (33 ms and 50 ms). The pattern reversed at 100 ms SOA. Like in Experiment 1, these results provide converging evidence for facilitation followed by inhibition effect of the spatially relevant cues. However, the evidence of inhibition of cued locations was seen only in saccade latency, but not in choice rate and saccade deviation. The possible reasons for weak inhibition are discussed in the general discussion. Finally, the latency on IR invalid trials was faster than relevant invalid trials at 33 ms but the pattern was reversed at 50 ms SOA suggesting reduced capture by IR cues only at 33 ms SOA. Interestingly, the cues had no influence on the accuracy of saccades irrespective of their relevancy.

## General Discussion

We examined the influence of subliminal cues presented at spatially-relevant or irrelevant locations on constrained free-choice saccades. The relevancy of the cues was established based on spatial ACS. In Experiment 1, participants were asked to make a saccade to one of the target circles in the upper visual field or the lower visual field depending on the colour of the central circle. In Experiment 2, the instruction was to choose one of the target circles in the left or right visual field. In both the experiments, we observed that relevant subliminal cues influenced saccadic responses measured through choice rate, saccade endpoint deviation and saccade latency. Our objective was also to examine if and to what extent spatially-irrelevant cues influence saccadic decisions. In both the experiments, saccade latency data showed reduced capture by spatially-irrelevant cues in some conditions. Further, subliminal cues at IR cue locations lead to more errors in Experiment 1. These findings show that spatial ACS can modulate the influence of subliminal cues on constrained free-choice saccades.

### Time course of subliminal cueing of constrained free-choice saccades

The present results add to the growing evidence regarding the depths and limits of subliminal cueing of eye movements. We have shown that abrupt-onset subliminal cues can bias saccadic responses even when the saccade locations are chosen freely by the participants within some constraints. We expected the relevant subliminal cues to influence both the spatial (choice rate and saccade endpoint deviation) and temporal properties (latency) of the saccades. In both experiments, we observed a higher frequency of saccades to the (relevant) cued location at shorter SOAs (33 ms and 50 ms) which turned into inhibition at 100 ms SOA. A similar pattern was observed in the latency of the saccadic decisions. Participants were faster making saccades on relevant valid trials compared to the relevant invalid cue trials at short SOAs (33 ms and 50 ms). Inhibitory effects - defined by faster latency on relevant invalid cue trials compared to relevant valid trials were seen at longer SOA. The facilitatory effects obtained at the shorter SOAs are in confirmation with Huang et al. [15] results who also showed that subliminal cues can influence free saccadic decisions.

The pattern of facilitation at short SOA followed by inhibition at long SOA is a classic finding in studies with peripheral cues in a Posner cueing paradigm [8]. The “negative” effect typically observed in peripheral cueing at long SOA (> 100 ms) is known as the inhibition-of-return which arises due to the reluctance of the oculomotor system to revisit recently attended locations [51]. However, IOR is commonly observed in response time measures in Posner cueing tasks. In such tasks, participants are slower responding to targets when they are presented at the cued locations as opposed to the uncued locations at long SOAs. For the first time, we have shown an inhibitory effect of subliminal cues on both frequency and latency of constrained free choice saccades.

The existence of such inhibitory effects even when people freely make decisions about where to look is in line with the underlying explanation of IOR as a foraging mechanism of the visual system which helps individuals seek out new information. We acknowledge that the inhibition was not strong (and statistically significant) in all measures. This can probably be attributed to our relatively “short” long SOA. It is likely that we have captured only the beginning of the inhibitory effects and that strong inhibition will be observed at longer (> 150 ms) SOAs. The other possibility is that the inhibitory effects were weakened by the blocking of SOA. In a peripheral cueing study with double targets, Wang et al. [47] observed IOR at 50 ms only when the 50 ms SOA trials were intermixed with 600 ms SOA trials (Experiment 2), but not when 50 ms SOA trials were presented alone (Experiment 1). The authors suggest that the temporal uncertainty in the appearance of the targets could have lead participants to disengage attention faster from cued locations resulting in observable IOR in the mixed condition. In contrast, the incentive to disengage from the cued location was lower in Experiment 1 as the participants could reliably (temporally) predict the appearance of the target using the cue. We might have similarly observed stronger inhibitory effects if we had intermixed the SOA conditions. Thus, while blocking SOA might be preferable in motor priming studies, it may not be so while investigating attentional mechanisms, especially using eye movements. Future studies should note this and choose the appropriate design.

Our results show that subliminally presented stimuli in the visual field can also generate activity in the oculomotor priority map and guide oculomotor selection. We found converging evidence for this across different eye movement measures: choice rate, saccade latency and saccade endpoint deviation. This is in line with previous studies that have suggested that a saliency map can be constructed without full awareness of the corresponding objects [56]. Pop-out without awareness demonstrated by Hseih et al. [56] is one such example where participants were presented with a display containing a feature singleton among homogenous distractors. The entire display was masked from awareness, but participants performed better on an orientation discrimination task at the location of the feature singleton than at other locations. This suggests that the location of the feature-singleton pops out and grabs attention without participants’ awareness. This was possible because the feature-singleton altered the saliency map even though it was suppressed from awareness. These results contribute to our growing understanding of the factors that guide overt visual selection.

### The role of spatial ACS in subliminal cueing of free-choice saccades

To our knowledge, this study is the first to examine the role of spatial ACS on subliminal cueing of free saccades. We had hypothesised that if spatial ACS can modulate the influence of irrelevant cues, saccade latencies on trials with irrelevant invalid cues should be faster than relevant invalid cue condition at short SOA. In contrast, if the spatial ACS fails to filter out the irrelevant cues, then the performance on IR invalid cue trials should be comparable to or worse than the relevant invalid cue condition. In Experiment 1, participants were faster in the presence of IR invalid cues at shorter SOAs and slower at long SOAs. Thus, cues at irrelevant locations did not capture attention as much as a relevant invalid cue. This suggests that the spatial ACS modulated the responses on IR cue trials. We observed this effect in Experiment 2 as well but only at 33 ms SOA. The accuracy data from Experiment 1 appears as an anomaly to the preceding argument. Participants made more errors on trials with IR cues compared to no cues suggesting that the IR cues captured attention causing more errors. An analysis of error saccades did not suggest that participants were more likely to make an error saccade to the IR cue location. Further, the effect of cue-relevancy on accuracy was seen only in Experiment 1. This indicates that the errors were more likely a result of the failure of the ACS in Experiment 1 rather than the irrelevant cues triggering eye movements. Thus, on trials where participants successfully managed to saccade to the correct locations, they were able to inhibit the IR cues.

This type of research has the potential to contribute to our understanding of the role of top-down attention on subliminal visual processing. They also help move forward the debate regarding the relationship between attention and consciousness. Showing that top-down attention can be directed at stimuli below the threshold of awareness suggests that attention does not necessarily require consciousness. Such dissociations can also inform us of the functional role of consciousness. While conducting such investigations, it is important to examine various domains of attention. As discussed in the introduction, several studies have shown that feature-based top-down attention can modulate the extent of subliminal processing [57, 38, 58]. In our study, we find some evidence to suggest that location-based top-down attention was able to reduce the influence of irrelevant subliminal cues. But, we acknowledge that the results with respect to the influence of irrelevant cues are not entirely conclusive. Since this is the first study to examine the influence of spatial ACS on subliminal cueing, we hope that these results will provide an impetus into more research on this topic which is necessary to evaluate the strength of these findings and arrive at robust conclusions.

It is possible to question if we would have observed stronger effects of spatial ACS if the trials had been blocked for each ACS. Previous studies examining the role of spatial ACS on attention capture with visible cues have administered trials with only one type of ACS to a participant [32, 33]. We did not have such blocking because many researchers have pointed out that top-down contingent capture effects seen in earlier studies could be explained through inter-trial priming effects arising due to blocking [eg., 59, 60, 61]. Thus, any top-down effects we might have observed could have been attributed to bottom-up inter-trial priming due to repeated occurrence of targets in particular locations. To account for this criticism, we only administered mixed sessions.

### Limitations

It is important to alert the readers to the fact that our no-cue condition is not the most appropriate baseline for measuring the influence of irrelevant cues. The abrupt-onset of a cue (irrespective of its location) also serves as an alerting signal to the visual system. This alerting mechanism is absent in the no-cue condition. For this reason, we did not compare the saccade latency on critical trials with no cue condition, which would have been a more appropriate comparison. This can be corrected by, for example, using an auditory tone on all the trials simultaneous to the presentation of the cue or no-cue [as used by 33].

Further, our use of the term "subliminal" might be called into question as the participants performed better than chance level in the cue localisation task possibly indicating that they were at least partially aware of the subliminal cues. It should be, however, noted that Bayesian analysis of Experiment 2 cue localisation indicated that there was anecdotal evidence to accept the null hypothesis. Nevertheless, the overall pattern of results in both Experiments was intriguing because we used 16 ms cues which are commonly used in subliminal cueing studies [21, 22, 23]. One possible reason why the cues could be localised better than chance is that the participants only had to detect the location of the cues. It is commonly observed in such studies that participants see “something” on the screen but can’t discriminate the exact identity of the cue. Thus, d’ is more likely to be at chance level when the cue visibility test involves some form of discrimination based on their identity rather than just detecting their presence. Since our cue visibility test was easy, participants might have been able to detect the cues with better than chance accuracy.

It is to be noted that several previous studies on subliminal cueing have similarly found above-chance performance on the cue visibility test [eg., 23, 27]. The cues are nevertheless argued to be subliminal in such cases sometimes for several reasons. First, participants in these studies often subjectively report to not have seen the cues. Second, the visibility index obtained on objective tests is considered to be an overestimation of the true awareness of the cues in the main experiment session. This is because the participants are explicitly informed about the nature of the cue and asked to pay attention to it in the visibility tests. However, they have no such explicit knowledge of the subliminal cues during the main experiment session. In line with this, some studies have found that the performance on the cue awareness test is independent of the cue’s influence on responses [62]. Third, it is impossible to rule out cueing effects in the visibility test itself. That is, the presence of the cues could have triggered responses associated with that location - similar to the cueing effects observed in the main experiment. Support for this “blindsight” like phenomenon comes from a recent study by Koivisto and Neuvonen [63] where participants performed a discrimination task and gave a subjective rating within a single response. The authors observed above-chance performance on the discrimination task even when participants reported to have seen “nothing” on the screen.

Given this, when can we truly say that participants are not conscious of certain stimuli? One of the major problems plaguing research on subliminal processing is the diversity of methods to induce and measure awareness [64]. While some people use objective visibility tests like the one we used, others rely on trial-wise subjective reports. In a paper comparing various subjective awareness measures, Sandberg, Timmermans, Overgaard, and Cleeremans [65] concluded that a graded perceptual awareness scale [PAS, 66] provided the strongest correlation between awareness and performance. As a consequence, graded subjective ratings, particularly PAS has been a popular method to elicit subjective reports of awareness and provide a better alternative to forced-choice discrimination tasks.

In sum, we acknowledge that we can not be confident that similar cueing effects on eye movements will be seen for cues that are completely invisible. However, like in many previous studies mentioned above, none of the participants verbally reported having “seen” the cues during the main experiment when questioned during a post-experiment briefing session. But, since we did not use a subjective awareness scale on a trial-to-trial basis, we can’t entirely rely on the verbal reports of the participants. Further studies with stricter control on the cue visibility are necessary to determine if the findings from our study can be generalised to subliminal cueing.

In conclusion, we have demonstrated that 1) subliminal cues can affect saccadic decisions even when these decisions are voluntary 2) Spatial ACS can modulate the influence of irrelevant cues under certain conditions. We observed this across two studies with different types of spatial relevancy. This study is a small contribution towards examining *if*, *when* and *how* attentional control can be exerted on capture by subliminal cues during free-choice eye movement behaviour. Such studies have the potential to contribute to our understanding of subliminal cueing as well as general theories of attention and eye movements.

### Ethics and Conflict of Interest

The author(s) declare(s) that the contents of the article are in agreement with the ethics described in http://biblio.unibe.ch/portale/elibrary/BOP/jemr/ethics.html and that there is no conflict of interest regarding the publication of this paper.

### Acknowledgements

The authors thank Ms. Vaishnavi Mohite and Mr. Aniruddha Ramgir for helpful discussions on this topic.

### Data availability statement

The data and materials for all experiments are available at https://osf.io/8nwdb/?view_only=41006770c1dc42efb5763dd065ec7c1e

## Appendix


