# Supplementary material for: To look or not to look: Subliminal abruptonset cues influence constrained free-choice saccades
Source: J Eye Mov Res. 2020 Jul 20;13(4):10.16910/jemr.13.4.2. doi: 10.16910/jemr.13.4.2 (PMC8004382; doi:10.16910/jemr.13.4.2)
Supplement: Supplementary file 1 [file jemr-13-04-b-SD1-01.pdf]

To look or not to look: Subliminal abrupt-onset cues influence constrained free-choice saccades

Seema Prasad & Ramesh Kumar Mishra

Center for Neural and Cognitive Sciences

University of Hyderabad, India

Supplementary material

Correspondence regarding this manuscript should be addressed to:

Seema Prasad, Center for Neural and Cognitive Sciences, School of Medical Sciences,

Science Complex, University of Hyderabad, Hyderabad, Telangana, India 500046

Ph No: 08886198575

Email id: gp.seema@gmail.com

The full outputs of the lme analysis of express saccades (80 ms < latency < 130 ms) are given

below. The analysis procedure was exactly same as that for regular saccades reported in the main text. For detailed information on the model specifications and variable coding, please refer to the data analysis section of the main text. Below we summarise the coding of different variables used as fixed effects.

**Variables**	**Coding**
IR_No	No cue: -1, IR cue: +1
R_No	No cue: -1, R cue: +1
RIV_RV	Relevant valid: -1, Relevant invalid: +1
RIV_IR	Relevant valid: -1, Irrelevant: +1
SOA100_50	50 ms: -1, 100 ms: +1
SOA100_33	33 ms: -1, 100 ms: +1
cueside (Experiment 1)	left: -1, right: +1
cueside (Experiment 2)	up: -1, down: +1
ACS signal (Experiment 1)	up: -1, down: +1
ACS signal (Experiment 2)	left: -1, right: +1

The formula used for each model and its output is give below. Note: $^{\#}p < 0.1$;*p<0.05; **p<0.01; ***p<0.001

Experiment 1

1. Frequency of saccades

```
d ~ ACSsignal_coded * soa50_33_coded + ACSsignal_coded * soa100_33_coded + (1 |
SubID) + (1 | serial)

==========================================================
                                   Dependent variable:
                              -----------------------------
                                            d
----------------------------------------------------------
soa100_33_coded                           0.069
                                        t = 0.661

ACSsignal_coded                           0.020
                                        t = 0.259
```

soa50_33_coded	0.064<br>t = 0.585
soa100_33_coded:ACSsignal_coded	-0.030<br>t = -0.289
ACSsignal_coded:soa50_33_coded	-0.232<br>t = -2.129*
Constant	0.055<br>t = 0.652
Observations	105
Log Likelihood	-129.075
Akaike Inf. Crit.	274.149
Bayesian Inf. Crit.	295.381

2. Saccade End location

```
xdev ~ ACSsignal_coded * cueside_coded * soa100_33_coded + ACSsignal_coded *
cueside_coded *  soa50_33_coded + (1 | SubID) + (1 | serial)
```

	Dependent variable: xdev	
	Relevant cues	Irrelevant cues
ACSsignal_coded	0.670<br>t = 2.004*	0.589<br>t = 1.848
cueside_coded	0.383<br>t = 1.170	-0.173<br>t = -0.564
soa100_33_coded	0.428<br>t = 1.082	0.570<br>t = 1.493
soa50_33_coded	-0.705<br>t = -1.487	-0.509<br>t = -1.112
ACSsignal_coded:cueside_coded	-0.395<br>t = -1.204	0.179<br>t = 0.581
ACSsignal_coded:soa100_33_coded	-0.199<br>t = -0.505	0.377<br>t = 0.993
cueside_coded:soa100_33_coded	-0.187<br>t = -0.479	0.407<br>t = 1.082
ACSsignal_coded:soa50_33_coded	0.260<br>t = 0.549	0.389<br>t = 0.870
cueside_coded:soa50_33_coded	0.272<br>t = 0.577	-0.370<br>t = -0.826
ACSsignal_coded:cueside_coded:soa100_33_coded	0.387<br>t = 0.980	-0.438<br>t = -1.165
ACSsignal_coded:cueside_coded:soa50_33_coded	0.921	0.671

	t = 1.954	t = 1.490
Constant	-0.701	-0.025
	t = -0.932	t = -0.032
Observations	684	702
Log Likelihood	-2,300.934	-2,360.604
Akaike Inf. Crit.	4,631.867	4,751.209
Bayesian Inf. Crit.	4,699.787	4,819.518

3. Saccade latency

```
Latency ~ ACSsignal_coded * conditionRIV_RV_coded * soa100_33_coded +
ACSsignal_coded  * conditionRIV_RV_coded *  soa50_33_coded + ACSsignal_coded  *
conditionRIV_IR_coded * soa100_33_coded + ACSsignal_coded *
conditionRIV_IR_coded * soa50_33_coded + (1 | SubID) + (1 | serial)
```

	Dependent variable:
	Latency
ACSsignal_coded	1.137
	t = 1.872
conditionRIV_RV_coded	-1.064
	t = -1.204
soa100_33_coded	-2.552
	t = -3.360***
soa50_33_coded	1.343
	t = 1.512
conditionRIV_IR_coded	-0.888
	t = -1.184
ACSsignal_coded:conditionRIV_RV_coded	-1.335
	t = -1.517
ACSsignal_coded:soa100_33_coded	0.204
	t = 0.270
conditionRIV_RV_coded:soa100_33_coded	1.014
	t = 0.920
ACSsignal_coded:soa50_33_coded	0.976
	t = 1.100
conditionRIV_RV_coded:soa50_33_coded	0.742
	t = 0.569
ACSsignal_coded:conditionRIV_IR_coded	0.909
	t = 1.215
soa100_33_coded:conditionRIV_IR_coded	0.833
	t = 0.880
soa50_33_coded:conditionRIV_IR_coded	0.228
	t = 0.209

```
ACSsignal_coded:conditionRIV_RV_coded:soa100_33_coded          2.196
                                                             t = 1.996*

ACSsignal_coded:conditionRIV_RV_coded:soa50_33_coded           0.350
                                                              t = 0.269

ACSsignal_coded:soa100_33_coded:conditionRIV_IR_coded         -0.835
                                                             t = -0.882

ACSsignal_coded:soa50_33_coded:conditionRIV_IR_coded           1.032
                                                              t = 0.944

Constant                                                      107.474
                                                           t = 138.480***

--------------------------------------------------------------------------------
Observations                                                    855
Log Likelihood                                               -3,511.161
Akaike Inf. Crit.                                             7,064.321
Bayesian Inf. Crit.                                           7,164.094
===============================================================================
```

4. Accuracy

```
d ~ soa100_33_coded * relevancyR_No_coded + soa100_33_coded *
    relevancyIR_No_coded + soa50_33_coded * relevancyR_No_coded +
    soa50_33_coded * relevancyIR_No_coded + (1 | SubID)
==================================================================
                                           Dependent variable:
                                       -----------------------------
                                                    d
------------------------------------------------------------------
soa100_33_coded                                   0.497
                                              t = 7.033***

relevancyR_No_coded                              -0.057
                                               t = -0.801

relevancyIR_No_coded                             -0.005
                                               t = -0.066

soa50_33_coded                                   -0.182
                                               t = -2.571*

soa100_33_coded:relevancyR_No_coded              -0.077
                                               t = -0.773

soa100_33_coded:relevancyIR_No_coded             -0.054
                                               t = -0.545

relevancyR_No_coded:soa50_33_coded                0.020
                                                t = 0.204

relevancyIR_No_coded:soa50_33_coded              -0.001
                                               t = -0.009

Constant                                          1.406
                                              t = 15.627***

------------------------------------------------------------------
Observations                                       189
Log Likelihood                                  -219.853
Akaike Inf. Crit.                                461.706
Bayesian Inf. Crit.                              497.365
```

```
==================================================================
```

Experiment 2

1. Frequency of saccades

```
d ~ ACSsignal_coded * soa50_33_coded + ACSsignal_coded * soa100_33_coded + (1 |
SubID) + (1 | serial)

==========================================================
                                  Dependent variable:
                             -----------------------------
                                           d
----------------------------------------------------------
soa100_33_coded                          0.138
                                       t = 1.406

ACSsignal_coded                          0.031
                                       t = 0.433

soa50_33_coded                          -0.068
                                      t = -0.689

soa100_33_coded:ACSsignal_coded         -0.161
                                      t = -1.641

ACSsignal_coded:soa50_33_coded           0.162
                                       t = 1.634

Constant                                -0.037
                                      t = -0.521

----------------------------------------------------------
Observations                              92
Log Likelihood                         -100.889
Akaike Inf. Crit.                       217.778
Bayesian Inf. Crit.                     237.953
==========================================================
```

2. Saccade End location

```
ydev ~ ACSsignal_coded * cueside_coded * soa100_33_coded + ACSsignal_coded *
cueside_coded *  soa50_33_coded + (1 | SubID) + (1 | serial)


===========================================================================
                                                   Dependent variable:
                                              ------------------------------
                                                          ydev
                                              Relevant cues    Irrelevant cues
---------------------------------------------------------------------------
ACSsignal_coded                                  0.759          0.601
                                               t = 2.130*     t = 1.698

cueside_coded                                   -0.164           0.258
                                               t = -0.460     t = 0.732

soa100_33_coded                                 -0.577           0.742
                                               t = -1.090     t = 1.466
```

soa50_33_coded	1.243	-0.191
	t = 2.444*	t = -0.372
ACSsignal_coded:cueside_coded	-0.131	0.210
	t = -0.367	t = 0.598
ACSsignal_coded:soa100_33_coded	0.270	1.346
	t = 0.543	t = 2.787**
cueside_coded:soa100_33_coded	0.871	0.273
	t = 1.774	t = 0.572
ACSsignal_coded:soa50_33_coded	0.083	-0.414
	t = 0.165	t = -0.822
cueside_coded:soa50_33_coded	-0.229	0.251
	t = -0.456	t = 0.496
ACSsignal_coded:cueside_coded:soa100_33_coded	0.377	-0.304
	t = 0.760	t = -0.636
ACSsignal_coded:cueside_coded:soa50_33_coded	-0.448	-0.066
	t = -0.878	t = -0.131
Constant	-0.943	-1.126
	t = -0.679	t = -0.810
Observations	441	427
Log Likelihood	-1,497.909	-1,448.814
Akaike Inf. Crit.	3,025.817	2,927.629
Bayesian Inf. Crit.	3,087.153	2,988.480

3. Saccade latency

```
Latency ~ ACSsignal_coded * conditionRIV_RV_coded * soa100_33_coded +
ACSsignal_coded * conditionRIV_RV_coded *  soa50_33_coded + ACSsignal_coded *
conditionRIV_IR_coded * soa100_33_coded +  ACSsignal_coded *
conditionRIV_IR_coded * soa50_33_coded + (1 | SubID) + (1 | serial)
```

	Dependent variable:
	Latency
ACSsignal_coded	-0.152
	t = -0.285
conditionRIV_RV_coded	-0.743
	t = -0.931
soa100_33_coded	-2.025
	t = -2.636**
soa50_33_coded	1.674
	t = 2.176*
conditionRIV_IR_coded	-0.273
	t = -0.405
ACSsignal_coded:conditionRIV_RV_coded	-1.089

```
                                                               t = -1.364

ACSsignal_coded:soa100_33_coded                                 -0.583
                                                               t = -0.792

conditionRIV_RV_coded:soa100_33_coded                            0.058
                                                                t = 0.053

ACSsignal_coded:soa50_33_coded                                   0.816
                                                                t = 1.071

conditionRIV_RV_coded:soa50_33_coded                            -0.403
                                                               t = -0.349

ACSsignal_coded:conditionRIV_IR_coded                            0.101
                                                                t = 0.149

soa100_33_coded:conditionRIV_IR_coded                           -0.226
                                                               t = -0.244

soa50_33_coded:conditionRIV_IR_coded                             1.037
                                                                t = 1.067

ACSsignal_coded:conditionRIV_RV_coded:soa100_33_coded            0.477
                                                                t = 0.435

ACSsignal_coded:conditionRIV_RV_coded:soa50_33_coded            -1.887
                                                               t = -1.635

ACSsignal_coded:soa100_33_coded:conditionRIV_IR_coded            1.260
                                                                t = 1.360

ACSsignal_coded:soa50_33_coded:conditionRIV_IR_coded            -0.519
                                                               t = -0.534

Constant                                                        106.161
                                                             t = 107.603***

---------------------------------------------------------------------------------
Observations                                                       868
Log Likelihood                                                 -3,554.550
Akaike Inf. Crit.                                               7,151.101
Bayesian Inf. Crit.                                             7,251.191
=================================================================================
```

4. Accuracy

```
d ~ soa100_33_coded * relevancyR_No_coded + soa100_33_coded *
    relevancyIR_No_coded + soa50_33_coded * relevancyR_No_coded +
    soa50_33_coded * relevancyIR_No_coded + (1 | SubID)

===================================================================
                                         Dependent variable:
                                    -----------------------------
                                                  d
-------------------------------------------------------------------
soa100_33_coded                                 0.265
                                           t = 3.598***

relevancyR_No_coded                             0.145
                                             t = 1.956

relevancyIR_No_coded                            0.003
                                             t = 0.036
```

soa50_33_coded	0.082
	t = 1.115
soa100_33_coded:relevancyR_No_coded	0.007
	t = 0.069
soa100_33_coded:relevancyIR_No_coded	0.201
	t = 1.929
relevancyR_No_coded:soa50_33_coded	-0.118
	t = -1.129
relevancyIR_No_coded:soa50_33_coded	-0.074
	t = -0.713
Constant	1.452
	t = 11.250***
Observations	159
Log Likelihood	-185.254
Akaike Inf. Crit.	392.508
Bayesian Inf. Crit.	426.266
